# Telemedicine in Long-Term Care Facilities During and Beyond COVID-19: Challenges Caused by the Digital Divide

**DOI:** 10.3389/fpubh.2020.601595

**Published:** 2020-10-26

**Authors:** Alexander Seifert, John A. Batsis, Anthony C. Smith

**Affiliations:** ^1^Center for Gerontology, University of Zurich, Zurich, Switzerland; ^2^School of Social Work, Institute for Integration and Participation, University of Applied Sciences and Arts Northwestern Switzerland, Olten, Switzerland; ^3^Division of Geriatric Medicine, Department of Nutrition, Center for Aging & Health, Gillings School of Global Public Health, University of North Carolina at Chapel Hill School of Medicine, Chapel Hill, NC, United States; ^4^Centre for Online Health, The University of Queensland, Brisbane, QLD, Australia; ^5^Centre for Health Services Research, The University of Queensland, Brisbane, QLD, Australia; ^6^Centre for Innovative Technology, University of Southern Denmark, Odense, Denmark

**Keywords:** older adults, telehealth, COVID-19, nursing care, telemedicine, long-term care

## Background

The COVID-19 pandemic has especially limited older adults from engaging in personal contact with others, as they have been classified as a high-risk population (1, 2). Increasing evidence shows that COVID-19 has taken a particularly heavy toll on older adults in long-term care facilities (LTCFs) (3). Older residents of LTCFs (e.g., nursing homes, retirement homes) often have daily care needs and are at especially high risk of COVID-19 due to the existence of multiple medical comorbidities and pre-existing conditions (4). As such, measures have often been put in place where such patients must shelter in place, maintain physical distancing from others during the pandemic and be subject to quarantine if they need to leave the facility for medical care. The context of living in LTCF means that older adults may be subject to even more protective measures that are administratively mandated, more so than the general population, including preventing their loved ones from visiting.

Telemedicine (also referred to as telehealth) has been recently shown to play an important role in distance-based treatment during this pandemic (5–8), despite the lack of quality, evidence-based trials that exist (9). Telemedical solutions are often feasible and acceptable in delivering care to older adults in LTCFs, even in those with sensory impairments such as hearing or visual loss (9). However, older adults are less likely than younger people to be able to take advantage of the opportunities enabled by modern information and communication technology (ICT) or telemedicine (10–14). Older adults living in LTCFs often (a) opt not to use the internet, (b) cannot afford internet access or ICT devices, (c) lack technical solutions with which to use telemedicine to connect virtually with doctors or other health professionals, (d) have physical or cognitive limitations that may limit possible telemedicine use or prevent them from using telemedicine at all without assistance, and (e) lack the skills to use ICT or telemedicine even if they do have access (9, 11, 15–18). Furthermore, the institutional may prevent the individual use of telemedicine; for example, individual use may depend on internet availability, ICT access, and telemedicine tools/software at a given facility. This article will outline and discuss the problems in this field and make recommendations for future discussion.

## ICT Use in Long-Term Care Facilities

While modern ICT use (such as the use of the internet, smartphones, and tablets) in healthy older adults has increased precipitously in recent years, the situation differs for those with multiple medical comorbidities and functional impairments and those with advanced age who are the primary residents of LTCFs (19–23). Seifert and Cotten (19) showed in their 2019 study that 21% of retirement home residents used the internet, 13% used a smartphone, and 5% used a tablet. Compared with non-users, internet users within LTCFs were more likely to be younger, healthier, and more functionally unimpaired (23, 24). The residents in this study (19) were also asked about their difficulties with modern technology with the statement, “Do you have difficulty operating modern technical devices?” Respondents rated the statement based on a 5-point Likert scale format (1 = “No, not at all” to 5 = “Yes, very much”). Among the respondents, 6.3% answered “No, not at all,” 10.1% answered “Not very much,” 26.9% answered “Partly,” 34.3% answered “Yes, somewhat,” and 22.5% answered “Yes, very much.” Schlomann et al. (22) recently conducted a study using data from North-Rhine–Westphalia, Germany, involving people aged 80 years and older living in private households and LTCFs. The researchers found that fewer than 3% of people in LTCFs used internet-connected ICT devices. ICT-device adoption was associated with the living environment and individual characteristics, including functional health, chronological age, education, and technology interest (22). These results indicate that individual characteristics and the living environment are both related to technology usage among the oldest age groups (21, 24).

## Telemedicine and Digital Infrastructure in Long-Term Care

Whether LTCF residents have access to using telemedicine is highly dependent on an underlying telemedicine infrastructure (e.g., internet availability, ICT access, telemedicine tools/software, ICT skills). The availability of modern ICT is limited within LTCFs, thus highlighting a significant deficiency in ICT infrastructure (25, 26). This deficit, in part, also includes a lack of technical skills among LTCF staff and potentially their apprehension of using technology within care facilities (27, 28), all inhibiting opportunities for telemedicine. The ongoing COVID-19 pandemic has prompted discussions of the positive outcomes of telemedicine for residents of LTCFs (29, 30). However, these discussions have also created awareness of the existing limitations of these facilities' current telemedicine infrastructures (11, 31).

Based on a Swiss representative national study (32) among managers of 466 LTCFs conducted in winter 2019, 14.6% of the LTCFs in Switzerland did not provide internet access to their residents. The survey was carried out as a standardized online survey of inpatient old-age homes throughout Switzerland. The respective managers were interviewed (32). Of the institutions that provided internet access, 66.3% offered residents an internet connection for free. The results show that basic internet access is not provided by every LTCF; however, Switzerland's ICT infrastructure and internet use of people aged 65 years and older are more equipped than other countries where individual residents need to pay for such services (33). Nevertheless, the results also show the degree of missing infrastructure for widespread telemedicine solutions within LTCFs (e.g., free internet access or mobile devices to use telemedicine apps privately).

The above-mentioned study (32) also asked the LTCF managers if they already used telemedicine (teleconsultation of doctors and/or health practitioners) within their facilities; only 3.9% of all 466 participating LTCFs used telemedicine. When asked if the managers evaluated telemedicine as useful for their facilities, 21.7% found it “rather useful” and 14.5% found it “very useful”; the rest (63.8%) found it rather not so useful or very non-useful. For this study, the authors did not have information related to the barriers or attitudes toward telemedicine; nevertheless, the authors demonstrated that telemedicine solutions were available in the minority among LTCFs in Switzerland, with few managers (36.2%) finding telemedicine useful. Only 11.1% of the managers in this study (32) said they involved their residents in decision-making about purchasing new technology for the institution. This corroborates the assumption that LTCFs are contextual settings with potential elements of a self-contained institution (34) with modest participation of residents in the process of initiating new technology solutions such as telemedicine.

## Recommendations

Based on the presented data, we recommend (1) education and training of staff and residents, (2) a solid telemedicine infrastructure, and (3) a system that promotes and integrates telemedicine in daily workflows within LTCFs.

First, given the rapid expansion of telemedicine, it is paramount to educate both LTCF staff and residents about how to use telemedicine, which could be useful in their daily lives during and beyond the current pandemic. The LTCF staffs are the coordinators and attend consultations with the patient; therefore, they are very important to include in all learning settings of telemedicine. It would be helpful to offer support and training to these people to increase their digital literacy skills. Establishing a workforce within LTCF environments with telemedicine competencies is important; this has not yet been anchored in education or evidence-based training (35). Learning new technical skills can even foster a certain sense of competence and autonomy (36) within older adults that can encourage the efficient use of other digital interventions. The special learning needs and cognitive resources of older adults need to be considered in these educational services, with attention paid to things such as the tempo of the learning session and the technological skill background of the older participants (37). These learning tools can be generally provided by LTCFs with the help of technical and management experts in telemedicine.

Second, besides the user side of telemedicine, the results from Switzerland reveal that LTCFs before, during, and probably beyond the COVID-19 pandemic have low levels of telemedicine infrastructure. This situation has pointed out that although telemedicine solutions would be ideal for medical treatments and consultations during physical distancing; however LTCFs are not yet ready for this task. It is critical to motivate developers and professional users (e.g., researchers, medical practitioners, and companies within the health sector) of telemedicine to take a closer look at how different designs and content can be tailored in a way that encourages trust and facilitates use among older people and LTCF staff. All stakeholders are encouraged to address these challenges and collaborate to promote the safe and evidence-based use of telemedicine during the current pandemic and future outbreaks (38, 39). The integration of end-users into workflows and the design process increases the usage and effectiveness of interventions, particularly as a partner in community-based participatory research in advance of developing a new digital intervention (40, 41). During any intervention, a real-time, support hotline, and contact partner can be used to assist the older participants when needed.

Third, telemedicine should not be system only used during a pandemic, but rather a routine method of providing services in our health system (31, 38, 42), and especially in LTCFs. We propose the following hurdles need to be overcome: (a) stable and high-performance internet access in all areas (cities or rural areas), (b) computers or mobile devices and software tools capable of engaging in telemedicine, (c) technical and software skills and skills in managing telemedicine processes among all stakeholders (e.g., residents, LTCF staff, doctors, medical staff), (d) willingness of all stakeholders to practice telemedicine, (e) interoperable communication systems and systems of exchange of health-related information and data, (f) availability of telemedicine support for staff or time for staff to do this within the daily business of care duties, (g) guidelines regarding the appropriate use of telemedicine, and (h) clinical and economical evidence from longitudinal studies within LTCF to support the effectiveness of the telemedicine services. Also, user focused studies are needed to better understand practical experiences from the perspective of resident and staff; and factors influencing uptake and acceptance in the health system.

Telehealth can be considered a “disruptive innovation process” by implying changing the way we provide service delivery. The importance of managing this change process well cannot be overstated by including all of the stakeholders associated with successful telehealth are accounted for. One way to further the “digital connection during physical distancing” idea would be to not limit communication applications such as chatting or video-calling to doctors, but to use such tools also for connecting with friends and relatives. The pandemic has fostered the potential of those social tools for digital connections within LTCFs (43), so why not also use those tools to help residents connect with the world beyond LTCFs? Current projects (44) use Skype, for example, for telemedicine under control for privacy and security requirements. However, also potential socio-economic inequalities in the use of telemedicine (45–47) or technology in general among older adults should be taken into account (15, 19, 33). Telemedicine enables cost savings (e.g., no transfer to the doctors' office), but also causes additional costs for older people (economical cost and acquired technical skills). Furthermore, potential barriers for digital excluded groups, such as older adults in LTCF, should be discussed and existing policy opinions should be considered when integrating telemedicine in everyday practice (48).

## Conclusions

The current pandemic highlights the challenges of providing LTCF residents with timely medical treatment during physical distancing and the potential of routinely using telemedicine in clinical care. Although the benefits of telemedicine have been widely reported, its routine use and its systematic evaluation for residents in LTCFs has been relatively limited. Integrating telemedicine is reliant on many complex and interrelated factors which must be addressed for successful adoption. Aside from the technical requirements, it is just as important to ensure that a supportive infrastructure are in place to support telemedicine services, systems are interoperable between service providers and recipients of care, staff are trained in its use, procedures are in place to ensure the safe and effective delivery of care, responsibilities for telemedicine care are clearly articulated, and funding is available to support the effort. The current pandemic has reminded us that innovative models of care that include telemedicine can be helpful, but organizational readiness to adopt telemedicine needs urgent attention.

## Author Contributions

All authors provided substantial contributions to this article from conception to final approval and share the same opinion.

## Conflict of Interest

The authors declare that the research was conducted in the absence of any commercial or financial relationships that could be construed as a potential conflict of interest.
